# Nitric oxide is involved in the cardioprotection of neonatal rat hearts, but not in neonatal ischemic postconditioning

**DOI:** 10.14814/phy2.16147

**Published:** 2024-08-04

**Authors:** Jan Doul, Marcela Minaříková, Zuzana Charvátová, Hana Maxová

**Affiliations:** ^1^ Department of Pathophysiology, Second Faculty of Medicine Charles University Prague Czech Republic; ^2^ Department of Physiology, Second Faculty of Medicine Charles University Prague Czech Republic; ^3^ Center for Experimental Medicine Institute for Clinical and Experimental Medicine Prague Czech Republic

**Keywords:** DEA‐NONO, ischemic postconditioning, L‐NAME, neonatal hearts, nitric oxide, SIN‐1

## Abstract

The cardioprotective effect of ischemic preconditioning (IPC) and ischemic postconditioning (IPoC) in adult hearts is mediated by nitric oxide (NO). During the early developmental period, rat hearts exhibit higher resistance to ischemia–reperfusion (I/R) injury, contain higher levels of serum nitrates, and their resistance cannot be further increased by IPC or IPoC. NOS blocker (L‐NAME) lowers their high resistance. Wistar rat hearts (postnatal Days 1 and 10) were perfused according to Langendorff and exposed to 40 min of global ischemia followed by reperfusion with or without IPoC. NO and reactive oxygen species donors (DEA‐NONO, SIN‐1) and L‐NAME were administered. Tolerance to ischemia decreased between Days 1 and 10. DEA‐NONO (low concentrations) significantly increased tolerance to I/R injury on both Days 1 and 10. SIN‐1 increased tolerance to I/R injury on Day 10, but not on Day 1. L‐NAME significantly reduced resistance to I/R injury on Day 1, but actually increased resistance to I/R injury on Day 10. Cardioprotection by IPoC on Day 10 was not affected by either NO donors or L‐NAME. It can be concluded that resistance of the neonatal heart to I/R injury is NO dependent, but unlike in adult hearts, cardioprotective interventions, such as IPoC, are most likely NO independent.

## 1 INTRODUCTION

The extent of ischemic injury depends not only on the intensity and duration of the ischemic insult, but also on the level of cardiac tolerance to ischemia. The tolerance can be increased by various cardioprotective interventions in adult hearts, such as ischemic preconditioning (IPC), ischemic postconditioning (IPoC), or remote ischemic preconditioning (RIPC) (see on review (Heusch, 2015)). These interventions affect cardioprotective pathways as mitochondrial permeability transition pore (mPTP) (Hausenloy et al., 2009), mitochondrial K‐ATP channels (mito‐K‐ATP) (Yang et al., 2004), reactive oxygen species (ROS) (Penna, Rastaldo, et al., 2006), various protective kinases (Tsang et al., 2004), and signaling molecules (e.g., adenosine (Morrison et al., 2007); nitric oxide (NO) (Alanova et al., 2015; Penna, Cappello, et al., 2006)). Blockade of any of these abolishes the positive effect of cardioprotective interventions in adult rat hearts (Matejikova et al., 2009). Also in humans, blockade of mPTP by cyclosporine at the time of reperfusion was associated with a smaller infarct (Piot et al., 2008). The beneficial effects of nitrite after intracoronary administration and in situ conversion to NO attenuates consequent myocardial reperfusion injury in the adult patients (Jones et al., 2015). Although theoretical knowledge is increasing, translation into clinical medicine is still problematic (see on review (Heusch, 2024)).

During ontogenetic development, the timeline of cardiac tolerance to ischemia shows a biphasic pattern in rats (Ostadal et al., 1999), the initially high resistance at birth declining during first 10 days of postnatal life, then increasing up to the end of the weaning period and then declining in males but remaining unchanged in females (Ostadal et al., 2009). Female sex hormones appear to be linked to NO pathway in mice, treatment with estrogen show increased eNOS activity and decreased vascular leukocyte accumulation after ischemia and reperfusion injury, this protective effect was abolished in the presence of PI(3)K or eNOS inhibitors (Simoncini et al., 2000). They are also protective even against some comorbidities that led to the loss of cardioprotection in male rat hearts (Babiker et al., 2019). However, Lieder et al. showed that sex is no determinant of IPC‐ and RIPC‐induced cardioprotection in isolated Lewis rat hearts (Lieder et al., 2019). These conflicting results shows complexity underlying the mechanisms involved in sex‐related differences in cardiac tolerance to ischemia. Protective effects of estrogen have many potential therapeutic implications, but in postmenopausal women may actually increase the incidence of IHD (Ostadal & Ostadal, 2014).

There are few publications regarding the increased tolerance of neonatal hearts to I/R injury. It turns out, however, that this issue is increasingly relevant in the field of pediatric cardiology or congenital heart surgery (Tan et al., 2018). In the neonatal rat heart mitochondria are more resistant to mPTP opening (Milerova et al., 2010); however, they do not differ from adult hearts in the amount of the key mPTP regulatory protein, cyclophilin D (Cyp‐D) (Milerova et al., 2016; Ostadal et al., 2019). Cardioprotective interventions were not able to increase the already high tolerance to ischemia in 1‐day‐old neonatal rat hearts, and their protective effect was usually observed only during the second postnatal week (Doul et al., 2015, 2019; Ostadalova et al., 1998, 2002). Moreover, 5‐hydroxydecanoate, a blocker of mito‐K‐ATP channels, failed to affect neonatal rat heart resistance to I/R injury and both IPC and IPoC in 10‐day‐old hearts (Doul et al., 2019; Ostadalova et al., 2002). Our study did not find differences in 3‐nitrotyrosine content in neonatal hearts, ruling out the involvement of superoxide in the mechanism of high resistance of the rat neonatal heart to I/R injury (Doul et al., 2019).

On the contrary, a study on isolated pups cardiomyocytes (Sanghvi et al., 2022) showed that inhibition of BKCa channels has a protective effect against myocardial ischemia and reperfusion injury. There is also evidence of the possible involvement of NO in the cardioprotection of neonatal rat heart. The high resistance of 1‐day‐old neonatal hearts was abolished by a NOS blocker (L‐NAME) that did not have any effect on 10‐day‐old hearts (Ostadalova et al., 2002). Our previous study found significantly higher levels of serum nitrates in 1‐day‐old animals compared to 10‐day‐old animals (Doul et al., 2019). These results perfectly correspond with the timing of actual decrease in cardiac tolerance to I/R injury (also between postnatal Days 1 and 10).

The aim of our study was therefore to determine the effect of NO donors and blockers in the neonatal period (1‐ and 10‐day‐old hearts) and on cardioprotective intervention IPoC in neonatal rat hearts. A pure NO donor (diethyamine‐nonoate; DEA‐NONO) and combined NO/superoxide donor (3‐morpholinosydnonimine; SIN‐1, used in case peroxynitrite plays role) were used as NO agonists and tested to evaluate their effect on cardioprotection. The nitric oxide synthase (NOS) inhibitor L‐NAME was used to evaluate the effect of the blockade of NO. Finally, our study tested the effect of both NO donors and blockers on IPoC in 10‐day‐old hearts.

## 2 METHODS

### 2.1 Animals

A total of 158 neonatal Wistar rats aged 1 and 10 days of both sexes were used throughout the experiments. Consistent with previous studies (Ostadal et al., 2009) and our own observations, sex differences are not present in early postnatal development. Experimental and control groups were composed from at least two different litters. All mothers had free access to water and a standard laboratory diet (Altromin 1324, Velaz) ad libitum. Animals were housed in standard housing conditions on a 12‐h light/12‐h dark cycle.

### 2.2 Heart function evaluation

The animals were weighed, then killed by cervical dislocation. The chest was quickly opened and a stainless steel cannula (with an external diameter of 0.45 mm for 1 day old or 0.8 mm for 10 days old) was inserted into the ascending aorta. The heart was rapidly excised, and the atria were removed and the rest of heart was perfused in the Langendorff mode under constant pressure corresponding to the mean arterial blood pressure for the given developmental stage (Litchfield, 1958; Zicha et al., 1986), that is, 25 and 73 cm H_2_O on Days 1 and 10, respectively. The hearts were perfused with a Krebs–Henseleit solution containing (in mmol/l): NaCl 118.0; KCl 4.7; CaCl_2_ 1.25; MgSO_4_ 1.2; NaHCO_3_ 25.0; KH_2_PO_4_ 1.2; glucose 7.0 and mannitol 1.1. The solution was saturated by a mixture of 95% O_2_ and 5% CO_2_ (pH 7.4) and temperature was maintained at 37°C. The hearts were electrically stimulated at a rate of 200 beats/min using silver electrodes attached to the base of the heart. The stimulation was performed with pulses of alternating polarity, 1 ms duration and voltage set at 50% above the threshold level. The resting force was gradually increased by means of a micromanipulator to the level at which the developed force (DF) was approximately 80% of the maximum force reached at the optimum preload. The contractile function of the isolated heart was measured using an isometric force transducer connected by a glass fiber, two‐arm titanium lever, and silk suture (0.7 metric) to the apex of the heart. The DF (g) was evaluated automatically from the force signal transducer using PowerLab (LabChart software, ADInstruments) connected to the computer.

### 2.3 Experimental protocol for NO donors

To determine the actual concentrations of NO donors used in this study, their pharmacokinetics, and especially their half‐lives, need to be considered. Both of these substances mediate their effects via products of their decomposition rather than having a direct effect. According to Santa Cruz, the half‐life of SIN‐1 should be 1–2 h. Even more critical is DEA‐NONO, which should have a half‐life of 16 min (20°C) and possibly even less in higher temperatures (a Langendorff heart is perfused at 37°C). Although low concentrations of these substances have been reported to have an effect in Langendorff experiments, these studies did not describe their methods in detail (particularly solution preparation and storage before the experiment) (Grievink et al., 2016; Pasdois et al., 2013).

We decided to test both low and high concentrations of both of these chemicals (0.2 and 10 mg/L). The low concentration approximately corresponds to the previously cited studies, while the high concentration was selected based on a previous study evaluating the cardioprotective effect of SIN‐1, which evaluated multiple concentrations, to be “as high as possible” without causing toxicity (Lencova‐Popelova et al., 2016). The high concentration solutions were prepared and filled into the Langendorff apparatus immediately before the heart preparation. The low concentrations were prepared in advance in an aqueous solution which was immediately aliquoted and frozen at −20°C, then stored for no more than 3 days. Vials were unfrozen in a 37°C water bath just before the actual experiment. Although freeze and thaw cycle could theoretically affect the activity of substance, a study analyzing activities of different substances after a single freeze and thaw cycle found the differences to be negligible (Abraham et al., 2021).

Animals (1 and 10 days old) were killed and hearts were extracted as described before. After a period of stabilization, baseline values of DF were recorded. Hearts were perfused for 5 min with the NO donor, either DEA‐NONO (Sigma‐Aldrich, Cat#D184) or NO/superoxide donor SIN‐1 (Sigma‐Aldrich, Cat#567028) in a Krebs–Henseleit solution. At the beginning of reperfusion, the experimental hearts were perfused with identical solutions for another 20 min. Hearts were then reperfused with Krebs–Henseleit up to the maximum recovery of DF (the last value of DF before its decay). The control group (Controls) was perfused with Krebs–Henseleit solution for the entire period. DF was measured continuously during the reperfusion period in all hearts. Tolerance to ischemia was expressed as the recovery of DF (percentage of baseline values). At the end of the experiment, the hearts were weighed.

### 2.4 Experimental protocol for NO blockade

Animals (1 and 10 days old) were killed and hearts were extracted. After a period of stabilization, baseline values of DF were recorded. One group of hearts was perfused for 10 min with NOS blocker, L‐NAME (Sigma‐Aldrich, Cat#N5751) in 200 μM concentration (Ostadalova et al., 2002) in Krebs–Henseleit solution, while the control group was perfused with Krebs–Henseleit only. At the beginning of reperfusion, the experimental hearts were perfused with an identical solution for another 20 min. All hearts were then reperfused with Krebs–Henseleit up to the maximum recovery of DF (the last value of DF before its decay). The control group was perfused with Krebs–Henseleit solution for the entire period. DF was measured in all hearts continuously during the reperfusion period. Tolerance to ischemia was expressed as the recovery of DF (percentage of baseline values). At the end of the experiment, the hearts were weighed.

### 2.5 Experimental protocol for ischemic postconditioning

**FIGURE 1:**
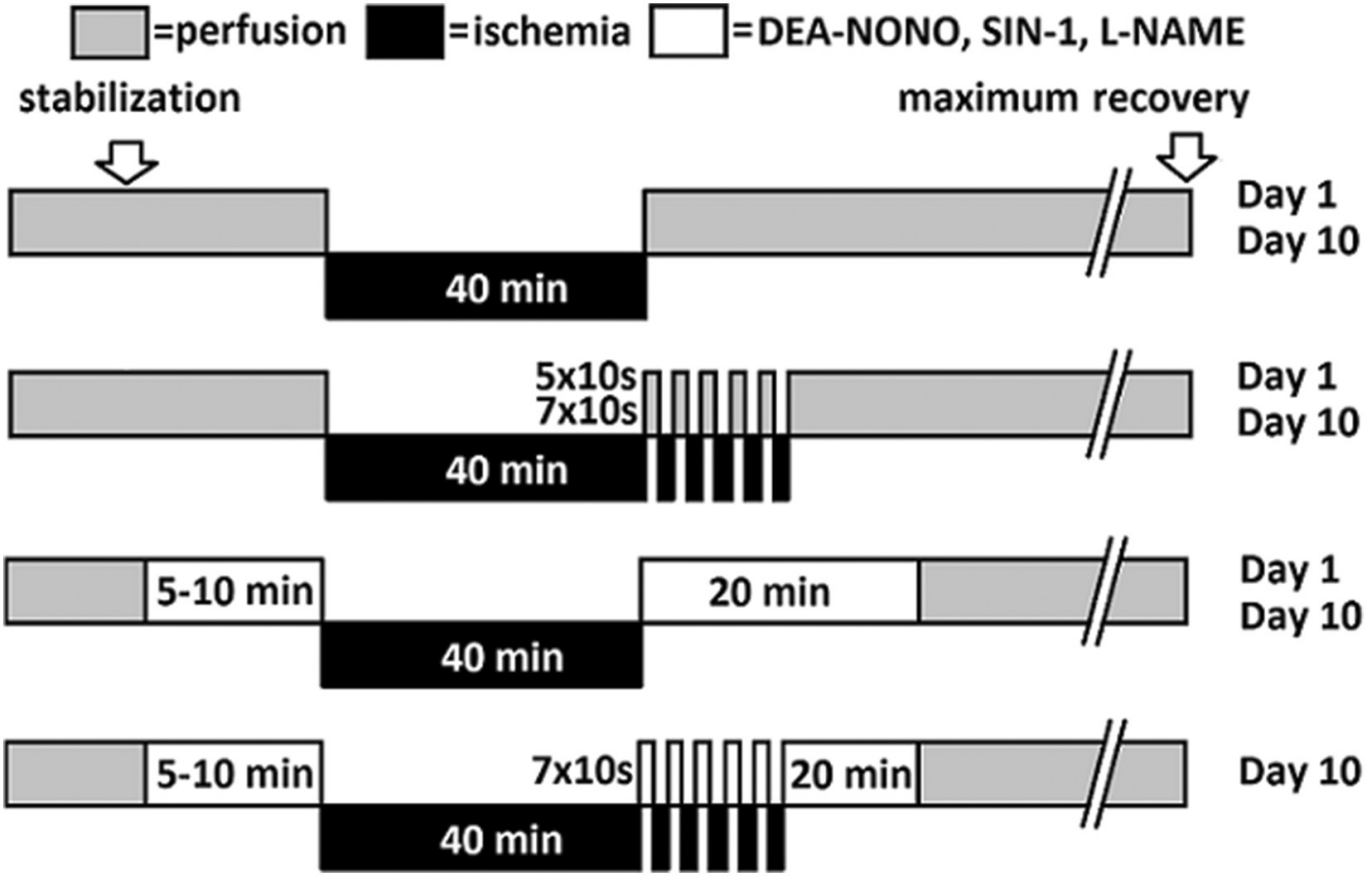
Scheme summarizing the experimental groups and ischemic postconditioning protocols.

Animals (10 days old) were killed and hearts were extracted. After a period of stabilization, baseline values of DF were recorded. All hearts were exposed to 40 min of global ischemia. At the beginning of reperfusion, one group of hearts was postconditioned by subjecting them to five or seven 10‐s periods of global ischemia (IPoC 5 × 10 s or IPoC 7 × 10 s), each separated by a period of reperfusion of the same duration (Figure 1). The control group was simply reperfused up to the maximum recovery of DF (the last value of DF before its decay). To test the effect of NO on IPoC, additional groups of hearts were subjected to IPoC (7 × 10 s) and DEA‐NONO or SIN‐1 or L‐NAME perfusion simultaneously. DF was measured in all hearts continuously during the reperfusion period. Tolerance to ischemia was expressed as the recovery of DF (percentage of baseline values). At the end of the experiment, the heart weight was recorded.

### 2.6 Statistical analysis

The results are expressed as means ± SD. Each observation was obtained from at least seven heart preparations in each group. Body weight, heart weight, baseline, and recovery of DF values were evaluated by one‐way ANOVA followed by the Student–Newman–Keuls test. The data for IPoC and effects of NO donors and blocker were also evaluated by one‐way ANOVA followed by the Student–Newman–Keuls test. Where two explanatory variables were considered simultaneously (treatment and day in Figure 4), the ANOVA model encoded all possible combinations of the two variables as a single categorical predictor. Severely arrhythmic hearts were excluded from analysis. Statistical analysis was performed in (R Core Team, 2023) using the package agricolae (De Mendiburu & Simon, 2015). The figures were created using GraphPad Prism 6.07 for Windows (Graph Pad Software, La Jolla California USA). The results were considered statistically significant when *p* < 0.05. The code and data for the statistical analysis are available in the Figshare repository (DOI:10.6084/m9.figshare.22682977).

## 3 RESULTS

### 3.1 Tolerance to ischemia

Body and heart weights and baseline contractile parameters are summarized in Table 1. The body and heart weights as well as DF increased between Days 1 and 10. Tolerance to ischemia significantly declined between Days 1 and 10.

**TABLE 1:** Morphometry and cardiac function parameters in 1‐ and 10‐day‐old animals.

Day	Protocol	*n*	Body weight (g)	Heart weight (mg)	HW/BW (mg/g)	DF (g)	DF rec. (%)
1	Controls	10	6.16 ± 0.71	34.56 ± 6.14	5.59 ± 0.60	0.53 ± 0.23	60.453 ± 12.998
	DEA‐NONO low	9	6.18 ± 0.59	32.59 ± 4.77	5.29 ± 0.72	0.44 ± 0.22	74.959 ± 16.616
	DEA‐NONO high	10	6.38 ± 0.32	37.16 ± 5.34	5.83 ± 0.81	0.49 ± 0.18	56.539 ± 23.720
	SIN‐1 low	7	5.96 ± 0.67	33.40 ± 3.77	5.62 ± 0.39	0.72 ± 0.32	56.209 ± 6.635
	SIN‐1 high	10	6.30 ± 0.51	32.14 ± 6.90	5.07 ± 0.83	0.49 ± 0.17	55.903 ± 19.420
	L‐NAME	7	6.17 ± 0.15	37.14 ± 4.58	6.01 ± 0.69	0.75 ± 0.34	44.926 ± 10.818
10	Controls	13	23.65 ± 2.21	117.09 ± 11.22	4.97 ± 0.47	2.09 ± 0.61	32.014 ± 8.074
	DEA‐NONO low	10	21.84 ± 2.21	121.36 ± 14.69	5.56 ± 0.37	2.30 ± 0.42	52.931 ± 10.619
	DEA‐NONO high	10	24.61 ± 4.00	117.42 ± 17.31	4.81 ± 0.50	1.72 ± 0.47	29.685 ± 9.372
	SIN‐1 low	9	20.06 ± 0.89	103.60 ± 7.80	5.14 ± 0.32	2.17 ± 0.25	42.861 ± 9.719
	SIN‐1 high	8	22.36 ± 1.00	115.23 ± 16.23	5.17 ± 0.78	1.63 ± 0.60	40.923 ± 10.658
	L‐NAME	9	22.42 ± 1.95	111.32 ± 11.57	4.98 ± 0.49	2.24 ± 0.35	54.908 ± 13.097
	IPoC 5 × 10 s	9	23.56 ± 2.19	114.97 ± 13.22	4.88 ± 0.31	2.27 ± 0.20	33.396 ± 7.389
	IPoC 7 × 10 s	9	23.31 ± 3.80	117.92 ± 21.98	5.07 ± 0.64	2.29 ± 0.44	45.372 ± 10.973
	IPoC + DEA‐NONO	11	19.99 ± 3.50	101.14 ± 12.17	5.11 ± 0.48	2.05 ± 0.22	48.995 ± 9.510
	IPoC + SIN‐1	9	21.72 ± 1.08	115.26 ± 6.94	4.96 ± 0.41	2.03 ± 0.48	40.622 ± 5.134
	IPoC + L‐NAME	8	21.41 ± 3.50	110.61 ± 10.90	5.27 ± 0.96	1.96 ± 0.57	52.324 ± 10.124

*Note*: Values are means ± SD.

Abbreviations: DF, developed force; HW/BW, heart weight to body weight ratio; SD, standard deviation.

### 3.2 Effect of low concentrations of NO donors on neonatal hearts

**FIGURE 2:**
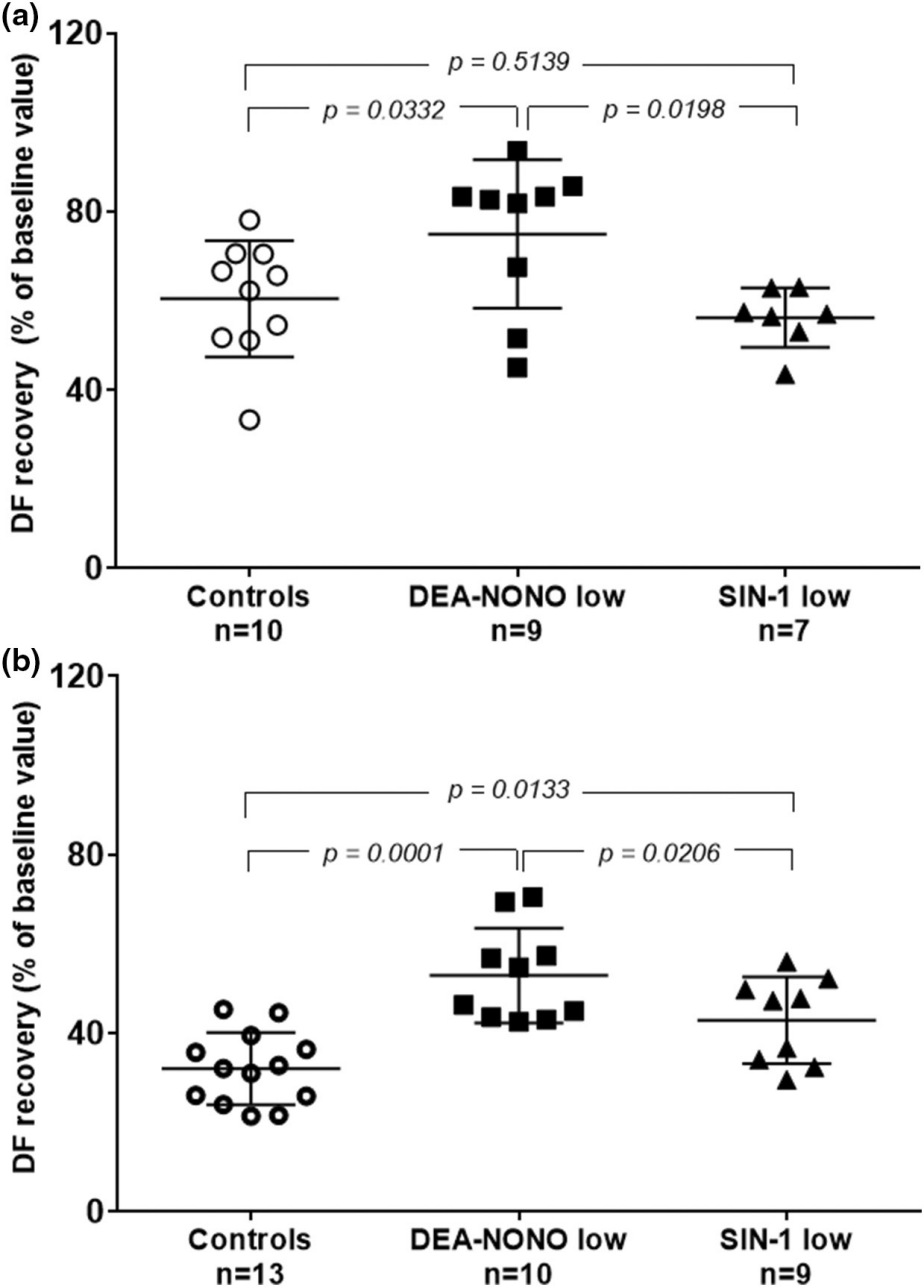
Cardioprotective effect of NO donors (DEA‐NONO and SIN‐1) in low concentrations (expressed as a percentage of recovery of DF baseline values) during early postnatal development (a) at Day 1, (b) at Day 10. ANOVA followed by the Student–Newman–Keuls test, data are represented as mean ± SD.

Low concentration of DEA‐NONO significantly increased the tolerance of neonatal hearts to I/R injury on both Days 1 (Figure 2a) and 10 (Figure 2b). Low concentration of SIN‐1 had a protective effect only on Day 10 (Figure 2b), but had no effect on Day 1 (Figure 2a). On Day 10, DEA‐NONO was significantly more protective compared to SIN‐1 (Figure 2b).

### 3.3 Effect of high concentrations of NO donors on neonatal hearts

**FIGURE 3:**
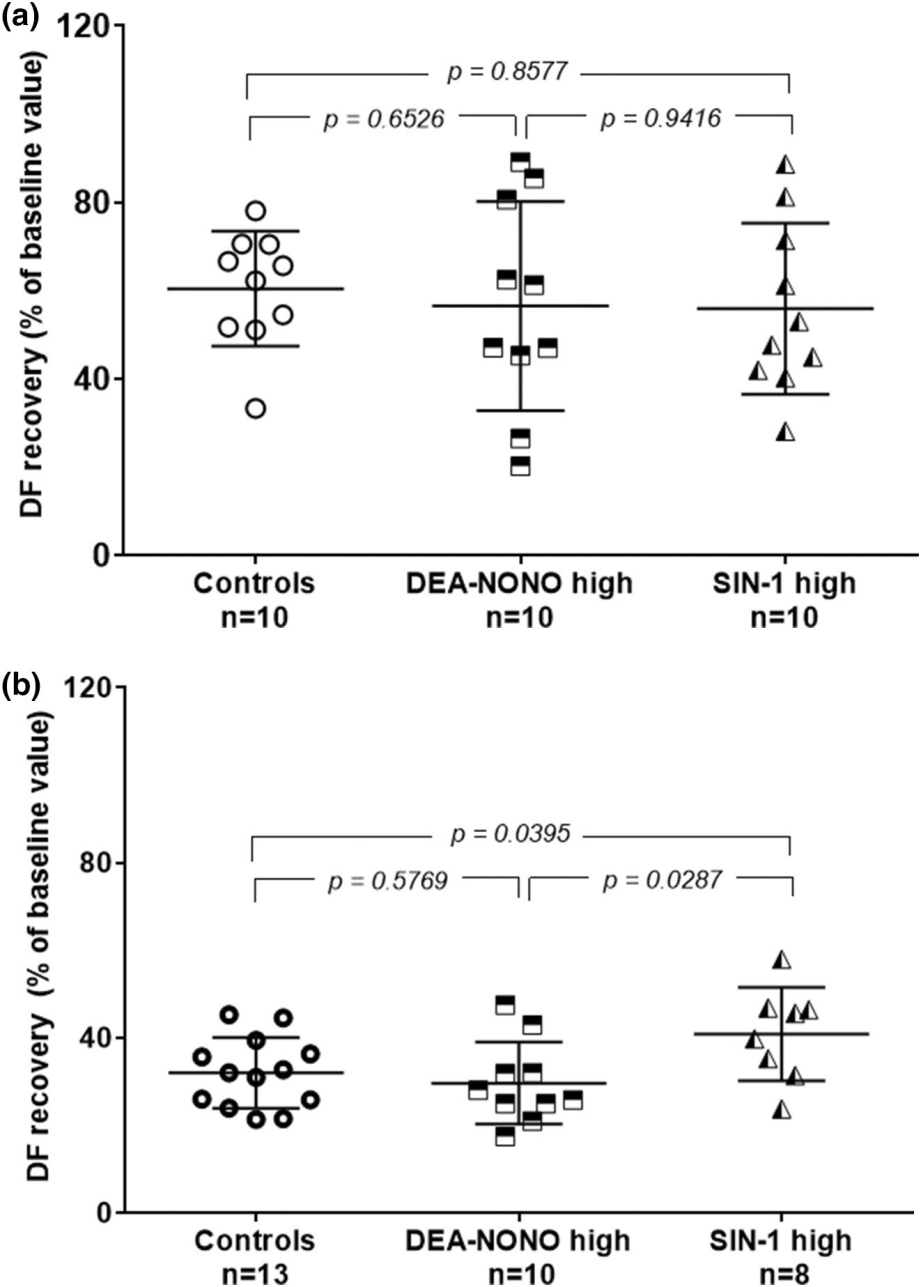
Cardioprotective effect of NO donors in high concentrations (expressed as a percentage of recovery of DF baseline values) during early postnatal development (a) at Day 1, (b) at Day 10. ANOVA followed by the Student–Newman–Keuls test, data are represented as mean ± SD.

High concentration of SIN‐1 significantly increased tolerance to I/R injury only on Day 10 (Figure 3b). High concentration of DEA‐NONO had no effect on either day (Figure 3).

### 3.4 Effect of NOS blocker L‐NAME on neonatal hearts

**FIGURE 4:**
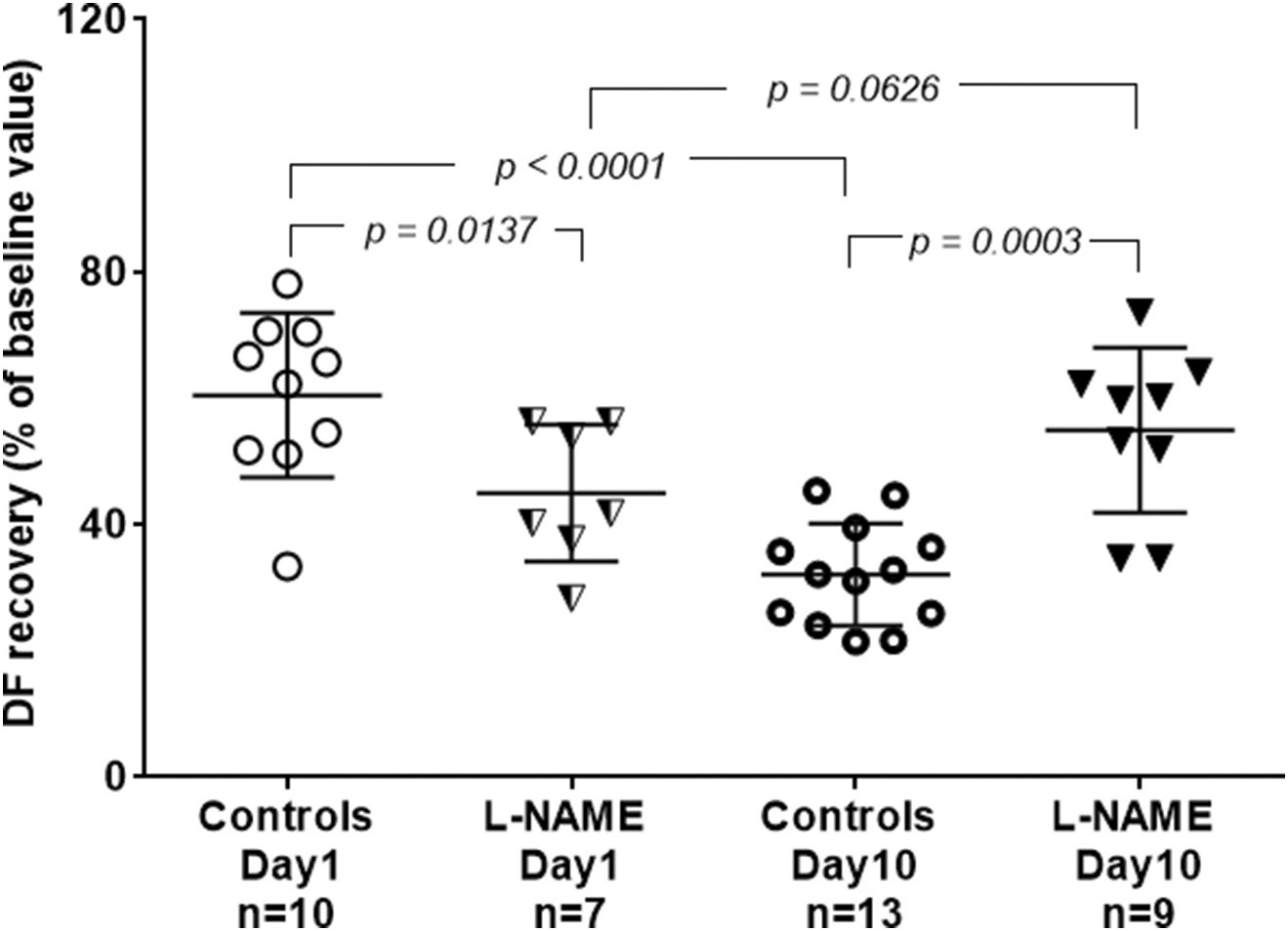
Effect of L‐NAME on cardioprotection (expressed as a percentage of recovery of DF baseline values) during early postnatal development. This figure also shows the significant difference between Days 1 and 10 controls. ANOVA followed by the Student–Newman–Keuls test, data are represented as mean ± SD.

In agreement with a previous study (Ostadalova et al., 2002), L‐NAME significantly reduced 1‐day‐old heart tolerance to I/R injury (Figure 4). However, on Day 10, L‐NAME significantly increased neonatal heart tolerance to I/R injury (Figure 4).

### 3.5 Effect of ischemic postconditioning on neonatal hearts

**FIGURE 5:**
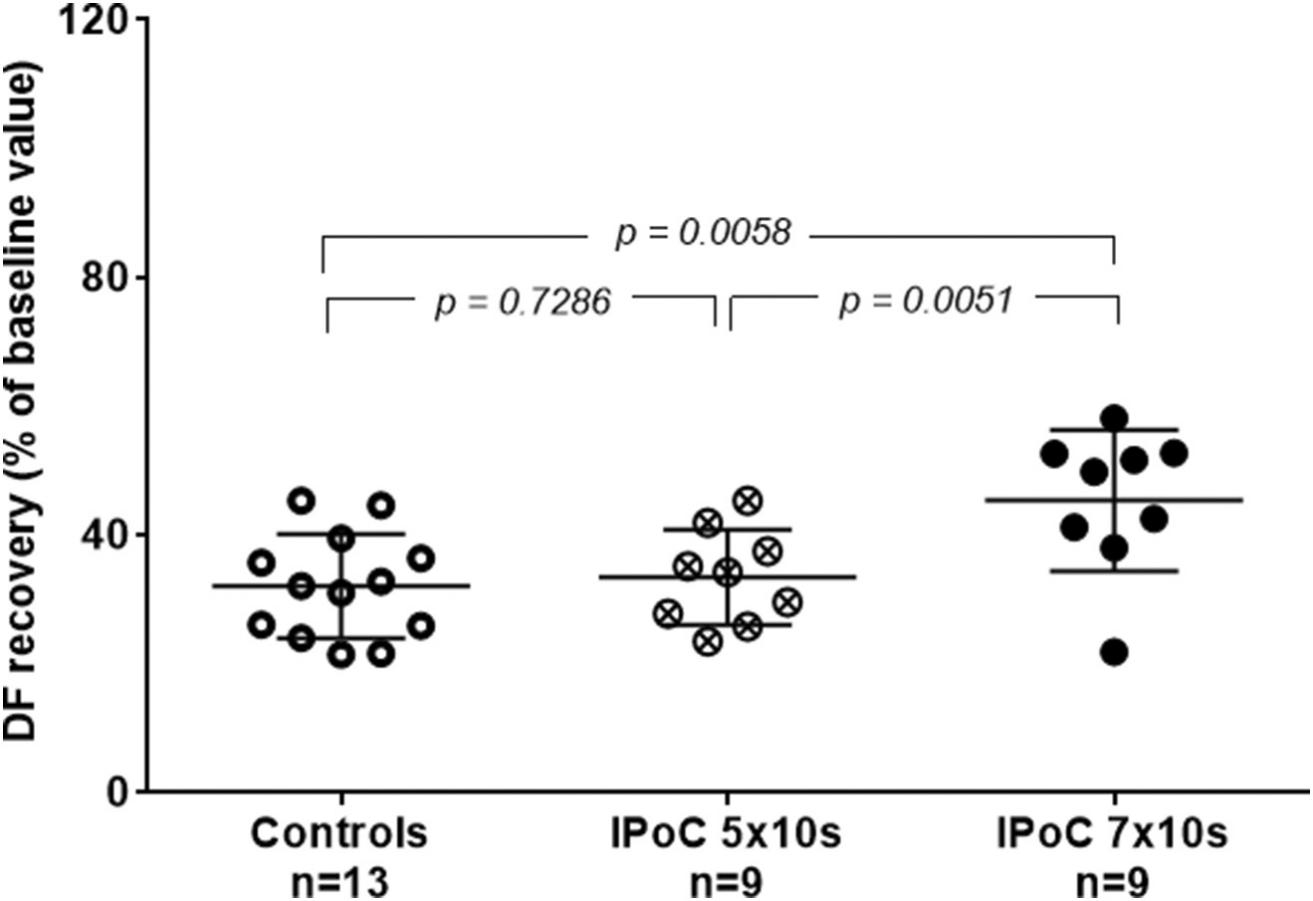
Cardioprotective effect of ischemic postconditioning (expressed as a percentage of recovery of DF baseline values) at Day 10. ANOVA followed by the Student–Newman–Keuls test, data are represented as mean ± SD.

IPoC protocol 5 × 10 s had no significant cardioprotective effect on Day 10 (Figure 5). However, increasing the number of postconditioning cycles to 7 × 10 s did restore the significant effect of IPoC on 10‐day‐old neonatal hearts (Figure 5).

### 3.6 Effect of NO donors and blockers on IPoC in neonatal hearts

**FIGURE 6:**
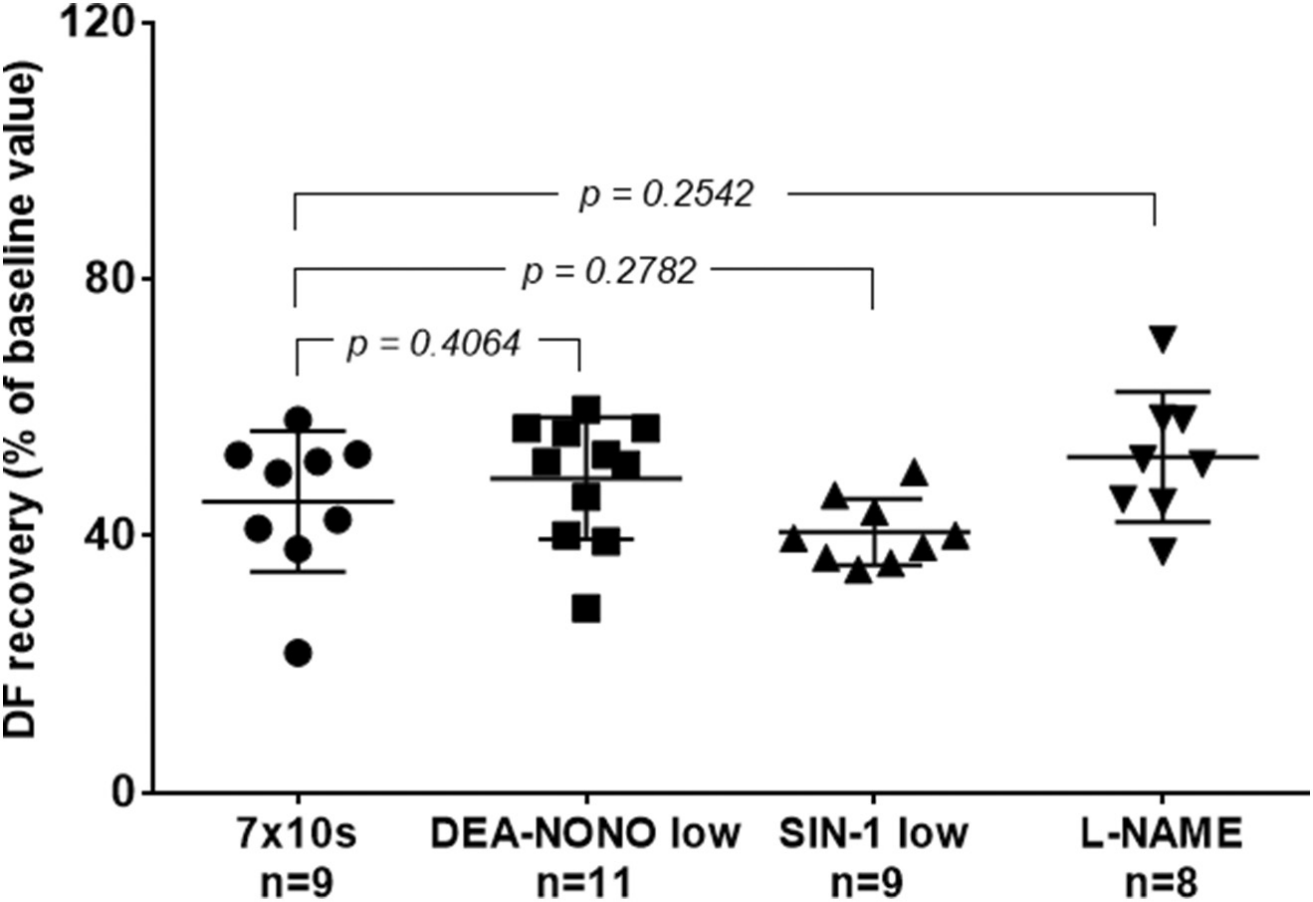
Effect of NO donors and L‐NAME on ischemic postconditioning (expressed as a percentage of recovery of DF baseline values) at Day 10. ANOVA followed by the Student–Newman–Keuls test, data are represented as mean ± SD.

The cardioprotective effect of IPoC (7 × 10 s) was not significantly affected by DEA‐NONO, SIN‐1 (low concentrations) or L‐NAME (Figure 6).

## 4 DISCUSSION

The confirmation of the cardioprotective effect of NO in neonatal heart is the main result of this study. Tolerance to ischemia on Day 1 was increased by DEA‐NONO in low concentration and decreased by L‐NAME, showing a possible dose‐dependent relationship between the actual content of NO in 1‐day‐old hearts and their resistance to I/R injury. This finding is further supported by the fact that 1‐day‐old animals have a higher amount of serum nitrates compared to 10‐day‐old animals, as determined by our previous study (Doul et al., 2019). On postnatal Day 10, where serum nitrates were found to be lower than on Day 1, the application of NO donor DEA‐NONO still increased their tolerance to I/R injury, showing a clear relationship between decrease of NO in neonatal hearts and their increased susceptibility to I/R injury. Taken together, these results strongly support the theory that NO is the actual substance responsible for the high resistance of neonatal hearts to I/R injury.

SIN‐1 was effective on Day 10, but significantly less than DEA‐NONO. We believe that this is because the actual cardioprotective molecule is NO and not superoxide. If products such as peroxynitrite were responsible for the cardioprotective effect in neonatal hearts, we would have expected SIN‐1 to create greater a protective effect than DEA‐NONO, however, that was clearly not the case. This is also in agreement with our previous study (Doul et al., 2019), where no difference was found in the amount of 3‐nitrotyrozine in neonatal hearts. These results demonstrate a possible difference between the cardioprotection of neonatal hearts and cardioprotective interventions in the adult hearts, which were ROS dependent (Penna, Rastaldo, et al., 2006), although additional experiments, such as using different ROS donors or different concentrations may be needed to definitively exclude the involvement of ROS in the cardioprotection of the neonatal heart. With the exception of SIN‐1 on Day 10, high concentrations of both NO donors failed to have any effect on cardioprotection in neonatal hearts. We cannot rule out a different threshold for the toxicity of these substances in neonatal hearts, and it is possible that they already reached toxic levels.

IPoC protocol 5 × 10 s failed to have any cardioprotective effect, but increasing the number of ischemia and reperfusion cycles to 7 × 10 s did restore the cardioprotection. In many previous studies, similar protocols were reported to have conflicting results (for a review, see Skyschally et al., 2009). The fact that the total length of IPoC plays a role, but at the same time periods of ischemia need to be short (10 s and not 30 s or 60 s), actually points to a known mechanism of I/R injury—calcium overload. IPoC reduces the total amount of calcium in coronary flow during early reperfusion period and preserves acidosis (for explanation of its cardioprotective effect see Ovize et al., 2010), which gives time for the intracellular metabolism to recover and produce ATP to remove calcium from the intracellular space before mPTP can open. Longer IPoC protocol further reduces the total amount of calcium available to the cells during the critical period of early reperfusion and reduction of calcium in perfusate is known to be protective (Reichelt et al., 2009). However, it should be mentioned that the calcium overload is a very complex phenomenon that even in the adult hearts was described in a large series of studies (Cao et al., 2006; Kaljusto et al., 2010; Reichelt et al., 2009; Shintani‐Ishida et al., 2012; Zhang et al., 2006), involves multiple mechanisms (including membrane NCX, sarcoplasmic reticulum and mitochondrial calcium uniporter), and it remains to be clarified whether the same mechanisms are also valid for neonatal hearts.

This may actually be linked to the most surprising result of our study, the cardioprotective effect of L‐NAME on Day 10. Given the fact that L‐NAME had an opposite effect on Days 1 and 10, we believe that two different underlying mechanisms must be involved. On Day 1, where NO content is high and is exerting its cardioprotective effect, blockade of NO actually increases I/R injury. However, on Day 10, further reduction of NO may not affect the cardiomyocyte at all (already low resistance to I/R injury). This does not, however, rule out the possibility that L‐NAME affects other tissues, such as blood vessels, where further reduction of NO would cause vasoconstriction, reducing coronary flow and creating a “postconditioning‐like” effect. While this may seem like pure speculation, there is actually evidence backing up this theory: gentle reperfusion also has cardioprotective effect (Okamoto et al., 1986). While we did not measure coronary flow (as we did not expect this result), a previous study by Ostadalova did so and found reduction in coronary flow after the administration of L‐NAME (see tab. 2 in Ostadalova et al., 2002).

The last result of our study suggests that IPoC in 10‐day‐old hearts is completely unaffected by NO. This implies a major difference between mechanisms of cardioprotective interventions in adult hearts (Penna, Cappello, et al., 2006) and in neonatal hearts. This conclusion is already backed up by three independent findings: first, neither L‐NAME nor NO donors showed any effect on IPoC on Day 10 in this study: second, L‐NAME had no effect on IPC as determined by Ostadalova: and finally, in this study the resistance of 1 day old hearts was increased by DEA‐NONO, but it was not increased by IPC or IPoC in either of our previous studies (Doul et al., 2015, 2019; Ostadalova et al., 1998, 2002). If IPC or IPoC were indeed producing NO in 1‐day‐old hearts, they would be increasing its tolerance to I/R injury. Since both mito‐K‐ATP channel and NO do not appear to affect IPoC in the neonatal heart, we believe that cardioprotective interventions must exert their effect on Day 10 via a different mechanism that should be independent of these components. This mechanisms could be the reduction of calcium overload, as its blockade appears to be sufficient to protect the cells even without the involvement of traditionally described cardioprotective pathways (Cao et al., 2006; Kaljusto et al., 2010; Reichelt et al., 2009; Zhang et al., 2006).

This study was focused on the involvement of NO in the cardioprotection of neonatal hearts, but other mechanisms could be responsible for the cardioprotection of neonatal hearts as well. mPTP is an important structure in cardioprotection, but as was already mentioned, the timeline of the resistance of neonatal hearts to mPTP opening and the reduction of their postnatal tolerance to I/R injury differs (Drahota et al., 2012). There is also the possibility that NO and mPTP are linked (NO may nitrosylate mitochondrial proteins (Sun et al., 2013)), affect mitochondrial respiration (compare (Chauvin et al., 2001) and (Jekabsone et al., 2003)) or activate cardioprotective pathways (Penna, Cappello, et al., 2006). The neonatal heart shows differences in at least some intracellular kinases, particularly GSK3β, the phosphorylation of which changes between birth and Week 2 (Liaw et al., 2013). Whether these changes are linked to NO remains to be clarified. However, it should also be mentioned that the same study did not find the analogical difference in the Akt kinase, further suggesting that the mechanisms of cardioprotection may be partially different in the neonatal hearts compared to the adult hearts. Although neonatal hearts are well known for their changes in calcium metabolism, the timeline of the sensitivity of neonatal hearts to calcium blockers differs from their resistance to I/R injury (Ostadalova et al., 1993). While this makes calcium metabolism unlikely to affect the resistance of neonatal hearts on Day 1, it of course does not rule out the possibility of cardioprotective interventions on Day 10 being based on the reduction of calcium overload. Recently, various other forms of cellular death, such as necroptosis, have been proposed to be involved in the I/R injury (review (Heusch, 2020)), but in our opinion it remains to be clarified, whether they truly represent distinct mechanisms independent of the already proposed cardioprotective pathways. Necroptosis has been demonstrated to interact with mPTP (Karch et al., 2015) and we are not aware of any data regarding these mechanisms of cardiomyocyte deaths in the neonatal hearts.

It should also be noted that while I/R injury is predominantly studied in association with ischemic heart disease in adult patients, it can also play a role in pediatric patients during cardiac surgery (Modi et al., 2002). Recent evidence suggests the possible benefit of cardioprotective interventions (remote ischemic conditioning) on postoperative recovery and length of hospital stay in pediatric patients undergoing congenital heart surgery (Tan et al., 2018). In adult experiments, combining cardioprotective intervention (IPC) with pharmacological protection (Cyclosporine A) may lead to a greater cardioprotective effect (Gonzalez Arbelaez et al., 2016). Pharmacological interventions could provide additional benefit due to their easy application and possibly additional protective effect.

## 5 CONCLUSION

It can be concluded that the resistance of neonatal hearts to I/R injury is NO dependent. The source of this NO production and possible targets remain to be determined. Unlike in adult hearts, the effect of cardioprotective interventions on Day 10 is most likely NO independent. This effect may possibly be mediated by reduction of calcium overload.

## FUNDING INFORMATION

This study was supported by the project National Institute for Research of Metabolic and Cardiovascular Diseases (Program EXCELES, Project No. LX22NPO5104)–funded by the European Union–Next Generation EU. Author H.M. is supported by the Ministry of Health of the Czech Republic, grant number: 20‐02‐00052.

## CONFLICT OF INTEREST STATEMENT

The authors have no relevant financial or non‐financial interests to disclose.

## ETHICS STATEMENT

All investigations conformed to the “Guide for the Care and the Use of Laboratory Animals”, published by the US National Institutes of Health. All procedures were approved by the Animal Studies Committee of the Second Faculty of Medicine, Charles University. This article does not contain any studies with human participants performed by any of the authors.

## Data Availability

The dataset supporting the conclusions of this article is available in the Figshare repository, [DOI:10.6084/m9.figshare.22682977, URL:https://figshare.com/articles/dataset/Labchart_recordings_for_Doul_et_al_Nitric_oxide_is_involved_in_the_cardioprotection_of_neonatal_rat_hearts_but_not_in_neonatal_ischemic_postconditioning_/22682977].
